# Towards algorithm auditing: managing legal, ethical and technological risks of AI, ML and associated algorithms

**DOI:** 10.1098/rsos.230859

**Published:** 2024-05-15

**Authors:** Adriano Koshiyama, Emre Kazim, Philip Treleaven, Pete Rai, Lukasz Szpruch, Giles Pavey, Ghazi Ahamat, Franziska Leutner, Randy Goebel, Andrew Knight, Janet Adams, Christina Hitrova, Jeremy Barnett, Parashkev Nachev, David Barber, Tomas Chamorro-Premuzic, Konstantin Klemmer, Miro Gregorovic, Shakeel Khan, Elizabeth Lomas, Airlie Hilliard, Siddhant Chatterjee

**Affiliations:** ^1^ Department of Computer Science, University College London, London WC1E 6EA, UK; ^2^ Holistic AI, London W1D 3QH, UK; ^3^ Cisco Systems, London EC2M 7EB, UK; ^4^ School of Mathematics, University of Edinburgh, Edinburgh EH9 3FD, UK; ^5^ The Alan Turing Institute, British Library, London NW1 2DB, UK; ^6^ Unilever, London EC4Y 0DY, UK; ^7^ University of Oxford, Oxford OX1 2JD, UK; ^8^ Centre For Data Ethics and Innovation, London, UK; ^9^ Institute of Management Studies, Goldsmiths, University of London, London SE14 6NW, UK; ^10^ Department of Computing Science, University of Alberta, Edmonton, Alberta T6G 2H1, Canada; ^11^ Royal Institution of Chartered Surveyors, London SW1P 3AD, UK; ^12^ Ainstein AI, London, UK; ^13^ School of Social Sciences and Technology, Technical University of Munich, 80539 Munich, Germany; ^14^ St Pauls Chambers, Leeds LS1 5JF, UK; ^15^ Resilience Partners, London W1G 8QE, UK; ^16^ Columbia University, New York, NY 10027, USA; ^17^ ManpowerGroup, Milwaukee, WI 53212, USA; ^18^ University of Warwick, Coventry CV4 7AL, UK; ^19^ London Stock Exchange, London, UK; ^20^ UK HMRC, London, UK; ^21^ ValidateAI, London, UK

**Keywords:** artificial intelligence, machine learning, explainability, auditing, bias, transparency

## Abstract

­Business reliance on algorithms is becoming ubiquitous, and companies are increasingly concerned about their algorithms causing major financial or reputational damage. High-profile cases include Google’s AI algorithm for photo classification mistakenly labelling a black couple as gorillas in 2015 (Gebru 2020 In *The Oxford handbook of ethics of AI*, pp. 251–269), Microsoft’s AI chatbot Tay that spread racist, sexist and antisemitic speech on Twitter (now X) (Wolf *et al*. 2017 *ACM Sigcas Comput. Soc*. **47**, 54–64 (doi:10.1145/3144592.3144598)), and Amazon’s AI recruiting tool being scrapped after showing bias against women. In response, governments are legislating and imposing bans, regulators fining companies and the judiciary discussing potentially making algorithms artificial ‘persons’ in law. As with financial audits, governments, business and society will require algorithm audits; formal assurance that algorithms are legal, ethical and safe. A new industry is envisaged: Auditing and Assurance of Algorithms (cf. data privacy), with the remit to professionalize and industrialize AI, ML and associated algorithms. The stakeholders range from those working on policy/regulation to industry practitioners and developers. We also anticipate the nature and scope of the auditing levels and framework presented will inform those interested in systems of governance and compliance with regulation/standards. Our goal in this article is to survey the key areas necessary to perform auditing and assurance and instigate the debate in this novel area of research and practice.

## 1. Introduction

With the rise of artificial intelligence (AI), legal, ethical and safety implications of its use are becoming increasingly pivotal in business and society. We are currently entering a new phase of the ‘digital revolution’ in which privacy, accountability, fairness, bias and safety are becoming research priorities and debate agendas for engineering and the social sciences [1,2].

Like the ‘Big Data’ wave, we conceptualize this new phase of algorithmic decision-making and evaluation (Big Algo) using the 5V’s methodology [3]:

—
*Volume* : as resources and know-how proliferate, soon there will be ‘billions’ of algorithms.
— *Velocity*: algorithms making real-time decisions with minimal human intervention;
— *Variety*: from autonomous vehicles to medical treatment, employment, finance, etc.;
— *Veracity*: reliability, legality, fairness, accuracy and regulatory compliance as critical features.
— *Value*: new services, sources of revenue, cost-savings and industries will be established.

While in the last decade the focus was on ‘data protection’, there has now been a shift towards ‘algorithm conduct’. As a result, new technologies, procedures and standards will be needed to ensure that ‘Big Algo’ is an opportunity and not a threat to governments, business and society at large.

**Figure 1.**
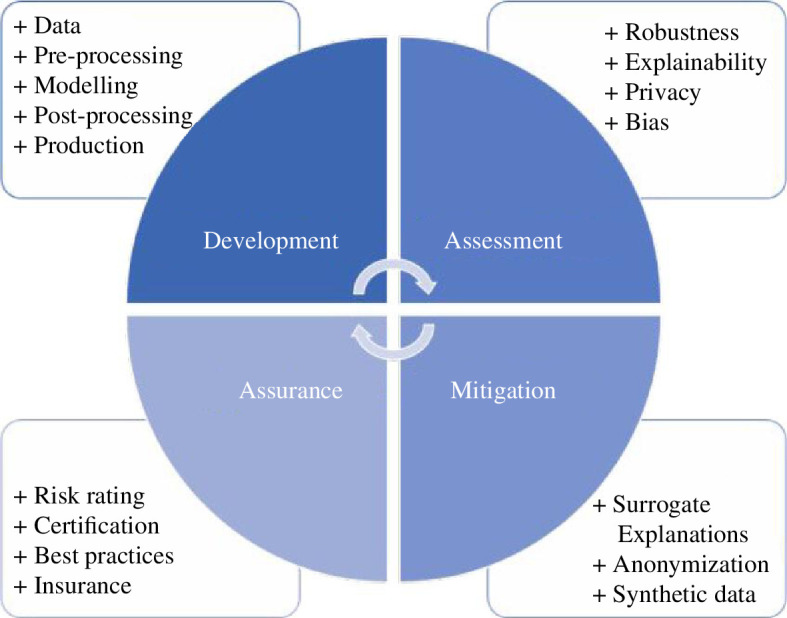
Dimensions and examples of activities that are part of algorithm auditing.

We conceptualize algorithm auditing as the research and practice of assessing, monitoring and assuring an algorithm’s safety, legality and ethics by embedding appropriate socio-technical interventions to manage and monitor risks it may be associated with. This practice encompasses current research in areas such as AI fairness, explainability, robustness and privacy, as well as matured topics of data ethics, management and stewardship. As with financial audits, governments, business and society will eventually require algorithm audits, that is, the formal assurance that algorithms are legal, ethical and safe. In a snapshot, figure 1 outlines the dimensions and examples of activities that are part of algorithm auditing. We define each one below.

— *Development*: the process of developing and documenting an algorithmic system.
— *Assessment*: the process of evaluating the algorithm’s behaviour and capacities.
— *Mitigation*: the process of servicing or improving an algorithm’s outcome.
— *Assurance*: the process of declaring that a system conforms to predetermined standards, practices or regulations.

A new industry, Auditing and Assurance of Algorithms and Data, is envisaged, with the remit to professionalize and industrialize AI, ML and associated algorithms. As with financial audits, we envisage that algorithm auditing will increasingly become a legal requirement as the AI regulatory landscape continues to evolve. However, granular technical knowledge is required to underpin these regulatory and legal frameworks seeking to promote AI auditing and assurance to ensure they are appropriate, robust and actionable.

Our goal with this article is to further the discourse in this novel area of algorithm audits, particularly with a view to contributing to, and challenging, emerging AI policy debates. We start by presenting a high-level overview of the key components that cover algorithm auditing in §3, namely algorithms, verticals of auditing, levels of access and mitigation. In §§4–7, we then provide a deeper exploration of algorithms, the verticals identified in §3, the seven levels of access defined in the literature and mitigation strategies for each risk vertical. We end with an overview of assurance processes concerning general and sector-specific processes, governance processes and how risks can be monitored, as well as discussing the potential for certification and insurance before we suggest avenues for further exploration. The purpose of this article is to (i) conceptualize a novel framework for conducting audits and mitigating risks; (ii) contribute to, and challenge, emerging policy debates emerging in this space; and (iii) inspire conversation around best practices for algorithm auditing among multidisciplinary stakeholders including academics, AI developers and deployers and regulators.

## 2. Key components of algorithm auditing

In this section, we describe the key parts encompassing algorithm auditing, namely the algorithm as the centrepiece of the process, the main verticals of auditing, ways to perform auditing and what happens subsequently, and finally, possible outcomes of auditing, namely algorithm assurance processes.

### 2.1. Object of audit: algorithms

An algorithm is a finite sequence of well-defined, computer-implementable instructions, typically to solve a class of problems or to perform a computation [4]. The key constituents of an algorithm are

— *Data*: input, output and simulation environment;
— *Model*: objective function, formulation, parameters and hyperparameters; and
— *Development*: design, documentation, building process and infrastructure and open-source libraries

In the 1980s and 1990s, expert systems—designed to simulate human decision-making using vast knowledge bases to solve problems [5]—were mainly in vogue and the main concern in relation to quality assurance was restricted to *Development and Model aspects* [6]. We should also mention that the focus during that period was more on accuracy and computational cost. Since the turn of the century, this paradigm has shifted—with most industrial applications of AI now relying on machine learning [7,8]. This shift towards sub-symbolic approaches (based on statistical learning methods) over symbolic representation [9] has added a new source of risk, namely *Data* (with model and data aspects interacting in a much more complex way than before), to the quality assurance process; discussions are now broadly around bias and discrimination, interpretability and explainability, privacy, with a reduced focus on performance and resilience of early systems.

### 2.2. What to audit: verticals of algorithm auditing

We conceptualize five stages underpinning the development aspect of algorithms [10–12] (see table 1):

— *Data and task setup*: collecting, storing, extracting, normalizing, transforming and loading data. Ensuring that the data pipelines are well structured, and the task (regression, classification, etc.) has been well specified and designed. Ensuring that data and software artefacts are well documented and preserved.
— *Feature pre-processing*: selecting, enriching, transforming and engineering a feature space.
— *Model selection*: running model cross-validation, optimization and comparison.
— *Post-processing and reporting*: adding thresholds, auxiliary tools and feedback mechanisms to improve interpretability, presenting the results to key stakeholders and evaluating the impacts of the algorithmic system on the business.
— *Productionizing and d*e*ploying*: passing through several review processes, from IT to business, and putting in place monitoring and delivery interfaces. Maintaining an appropriate record of in-field results and feedback.

**Table 1.** Interrelation between development stage and auditing verticals.

stage	explainability	robustness	fairness/bias	privacy
data and task setup	data collection and labelling	data accuracy	population balance	DPIA
feature pre-processing	dictionary of variables	feature engineering	fair representations	data minimization
model selection	model complexity	model validation	fairness constraints	differential privacy
post-processing and reporting	auxiliary tools	adversarial testing	bias metric assessment	model inversion
productionizing and deploying	interface and documentation	concept drift detection and continuous integration	real-time monitoring of bias metrics	rate-limiting and user’s queries management

Although these stages appear static and self-containing, in practice they interact in a dynamic fashion, not following a linear progression but a series of loops, particularly in between pre-processing and post-processing.

In table 1, we also list how each stage interacts with four key risk verticals, in line with some of the principles outlined by the European Commission in their white paper on AI excellence and trust [13], which we explore further in §5:

— *Privacy*: quality of a system to mitigate personal or critical data leakage.
— *Fairness/bias*: quality of a system to avoid unfair treatment of individuals or organizations.
— *Explainability (and interpretability)*: quality of a system to provide decisions or suggestions that can be understood by their users and developers.
— *Robustness*: quality of a system to be safe, not vulnerable to tampering.

In a similar fashion to the stages, each risk vertical—while appearing to be self-contained—also exhibits interdependencies. Though research on each vertical is mostly conducted in silos, there is growing consensus in the scientific and industry communities about the *trade-offs and interactions* between them. For example, accuracy, a component of robustness, may need to be traded for lowering any existing outcome metric of bias [14], making the model more explainable may affect a system’s performance and privacy [15,16], improving privacy may affect ways to assess adverse impacts of algorithmic systems [17] and so on. Optimization of these features and trade-offs will depend on multiple factors, notably the use-case domain, the regulatory jurisdiction, the risk appetite and values of the organization implementing an algorithm.

### 2.3. Ways to audit: levels of access for auditing

There are different levels of access that an auditor has during its investigation of an algorithm (see table 2). In scientific literature and technical reports, the prevalent common practice has been to categorize the knowledge about the system in two extremes: ‘white-box’ and ‘black-box’. In fact, the spectrum regarding the knowledge of a system is more of a continuum of ‘shades of grey’ than this simple dichotomy. This additional nuance allows for a richer exploration of the technologies available for assessment and mitigation, as well as the right level of disclosure that a certain business feels comfortable, or indeed might be legally required, to engage in.

**Table 2.** Landscape of algorithm auditing.

dimension	level 1 process access	level 2 model access (.)	level 3 input access *f*(*x*)	level 4 outcome access *f*(*x*), *y*	level 5 parameter control *f*_θ_(*x*), *y*	level 6 learning goal *L*(*f*_θ_(*x*), *y*)	level 7 ‘white-box’
explainability	checklist	feature relevance partial dependency	surrogate explanations	accuracy of explanations	stability of explanations	model complexity	documents and specific explanations
robustness	checklist	adversarial attacks	synthetic data	concept drift analysis	stability analysis	stress-testing	model selection and validation
fairness	checklist	adversarial fairness	bias in outcome	bias in opportunity	stability of bias metrics	trade-off of bias and loss metric	model selection and development
privacy	checklist	statistical disclosure	property and membership inference	inversion attacks	functionality stealing	model extraction	model security evaluation
information concealed	very high	high	high	high/medium	medium	medium	low
feedback detail	low	medium	medium	high/medium	high	high	very high
typical application	sales forecasting	cyber security	recruitment	credit-scoring	facial recognition	algorithmic trading	self-driving vehicle
appropriate oversight	guidelines	external auditing/ certification	external auditing	external auditing	external auditing	internal/external auditing	internal auditing

Hence, we can identify *seven levels of access* that an auditor can have to a system. It ranges from the highest level, that is, ‘white-box’, where all the details encompassing the model are disclosed, to the lowest level, that is, ‘process-access’ where only indirect observation of a system can be made. The levels in between are set by limiting access to the components behind the learning process (e.g. knowledge of the objective function, model architecture, training data, etc.). Level 7 contains all the assessment, monitoring and mitigation strategies of lower levels, with the report getting less detailed and accurate as levels decrease. Therefore, analysis and techniques requiring Level 7 cannot be used at Level 6 without proper assumptions and acceptable levels of inaccuracy.

### 2.4. After audit: mitigation strategies

Feedback received as an output of the audit interventions can be made to improve an algorithmic system’s outcome across the key verticals and stages. The more access to an algorithmic system, the more targeted, technical, diverse and effective will be the mitigation strategy. Table 3 lists possible interventions when ‘white-box’ access is provided. When the access available is lower than the ‘white-box’ level, some stages and procedures are omitted from this table (e.g. data and task setup or productionizing and deploying).

**Table 3.** Interrelation between development stage and mitigation strategies for ‘white-box’ access level.

stage	explainability	robustness	fairness/bias	privacy
data and task setup	dictionary of variables and datasheets	collecting targeted data, reframing loss function	alternative data sources	anonymization
feature pre-processing	avoiding excessive feature engineering	feature squeezing	synthetic data	dimensionality reduction
model selection	by-design interpretable models	adversarial training	counterfactual fairness	federated learning
post-processing and reporting	LIME, SHAP	high confidence predictions and confidence intervals	calibrated odds	model inversion mitigation
productionizing and deploying	recourse interface	‘circuit-breaking’	monitoring panels	rate-limiting and user’s queries management

### 2.5. Outcome of audit: assurance processes

The broader outcome of an auditing process is to improve confidence or ensure trust in the underlying system and then capture that through some certification mechanism. After assessing the system and implementing mitigation strategies, the auditing process assesses whether the system conforms to regulatory, governance and ethical standards. Providing assurance, therefore, needs to be understood from an interdisciplinary perspective, and measures need to be taken so that an algorithm’s trustworthiness can be exhibited. Below, we list key measures that embody the assurance process.

— *General and sector-specific assurance*: broad national regulation and standards (provided by organizations such as the National Institute of Standards and Technology (United States), the Information Commissioner’s Office (United Kingdom) and the European Union’s AI Act) with sectoral frameworks, such as in financial services (e.g. SEC, FCA, etc.), health (e.g. NIH, NHS, etc.) and real estate (e.g. RICS, IVS, USPAP)*.*
— *Governance*: from two aspects, namely technical assessments (robustness, privacy, etc.) and impact (risk, compliance, etc.) assessments.
— *Unknown risks*: discussing risk schemes and highlighting ‘red teaming’, which is used to mitigate unknown risks.
— *Monitoring interfaces*: outlining risk assessments and the use of ‘traffic-light’ user-friendly monitoring interfaces.
— *Certification*: numerous ways in which certification may occur, such as certification of a system or AI engineers.
— *Insurance*: a subsequent service to emerge as a result of assurance maturing.

Regulators face a growing challenge in both supervising the use of these algorithms among the sector(s) that they oversee and the use of algorithms in their own regulatory process via RegTech (Regulatory Technology) [18] and SupTech (Supervisory Technology) [19]. There are some other ‘soft’ aspects, related to the governance structure underpinning the development. These are related to defining an algorithm’s goals (e.g. what does it aim to achieve? How does it serve those it is making decisions about?). These could compose a statement of intention whereby the designer sets out a position statement in advance indicating what it is that the algorithm is supposed to do. This could facilitate judging whether the algorithm has performed as intended.

## 3. Algorithms

For completeness, this section unpacks algorithms across three domains: computational statistics (e.g. Monte Carlo methods), complex systems (e.g. agent-based systems) and AI and ML (e.g. artificial neural networks). While there may be some debate over the terminology, we find this classification helpful to distinguish between relatively well-established methods and more cutting-edge technologies.

— *Computational statistics*: computationally intensive statistical methods.
— *Complex systems*: systems with many interacting components whose aggregate activity is nonlinear and typically exhibit hierarchical self-organization under selective pressures.
— *AI algorithms*: mimicking a form of learning, reasoning, knowledge and decision-making.
  - Knowledge or rule-based systems
  - Evolutionary algorithms
  - Machine learning.

### 3.1. Computational statistics

Computational statistics models refer to computationally intensive statistical methods including resampling methods (e.g. bootstrap and cross-validation), Monte Carlo methods, kernel density estimation and other semi- and non-parametric statistical methods and generalized additive models [20,21]. Examples include:

— *Resampling methods*: a variety of methods for doing one of the following: (i) estimating the precision of sample statistics using subsets of data (e.g. jack-knifing) or drawn randomly from a set of data points (e.g. bootstrapping); (ii) exchanging labels on data points when performing significance tests (e.g. permutation tests); (iii) validating models by using random subsets (e.g. repeated cross-validation);
— *Monte Carlo methods*: a broad class of computational algorithms that rely on repeated random sampling to approximate integrals, particularly used to compute expected values (e.g. options payoff) including those meant for inference and estimation (e.g. Bayesian estimation, simulated method of moments);
— *Kernel density estimation*: a set of methods used to approximate multivariate density functions from a set of datapoints; it is largely applied to generate smooth functions, reduce outlier effects and improve joint density estimations, sampling and derive nonlinear fits;
— *Generalized additive models*: a large class of linear models widely used for inference and predictive modelling (e.g. time series forecasting, curve-fitting, etc.);
— *Regularization methods*: calibration techniques used to minimize loss and prevent overfitting and underfitting to make a model more generalizable. Regularization methods are increasingly used as an alternative to traditional hypothesis testing and criteria-based methods, for allowing better quality forecasts with many features.

### 3.2. Complex systems

A complex system is any system featuring a large number of interacting components (e.g. agents, processes, etc.) whose aggregate activity is nonlinear (not derivable from the summations of the activity of individual components) and typically exhibits hierarchical self-organization under selective pressures [22,23]. Examples include:

— *Cellular automata*: a collection of cells arranged in a grid, such that each cell changes state as a function of time according to a defined set of rules that includes the states of neighbouring cells;
— *Agent-based models*: a class of computational models for simulating the actions and interactions of autonomous agents (individual or collective entities such as organizations or groups) with a view to assessing their effects on the system as a whole;
— *Network-based models*: a complex network is a graph (network) with non-trivial topological features—that do not occur in simple networks such as lattices or random graphs but often occur in graphs modelling of real systems
— *Multi-agent systems*: this subarea focuses on formulating cooperative–competitive policies for a multitude of agents with the aim of achieving a given goal; this topic has significant overlap with reinforcement learning and agent-based models.

### 3.3. AI and machine learning

There are broadly two classes of AI algorithms, which might be termed: *static algorithms*—traditional programs that perform a fixed sequence of actions; and *dynamic algorithms*—that embody machine learning and evolve. It is these latter ‘intelligent’ algorithms that present complex technical challenges for testing and verification, which will impact and demand further regulation.

These algorithms span three main communities:

— Knowledge-based or heuristic algorithms (e.g. rule-based): where knowledge is explicitly represented as ontologies or IF–THEN rules rather than implicitly via code [5].
— Evolutionary or metaheuristics algorithms: a family of algorithms for global optimization inspired by biological evolution, using population-based trial and error problem solvers with a metaheuristic or stochastic optimization character (e.g. genetic algorithms, genetic programming, etc.) [24,25]
— Machine learning algorithms: a type of AI program with the ability to learn without explicit programming and can change when exposed to new data; mainly comprising *supervised* (e.g. support vector machines, random forest, etc.), *unsupervised* (e.g. K-means, independent component analysis, etc.) and *reinforcement learning* (e.g. Q-learning, temporal differences, gradient policy search, etc.) [7,8]. Russell & Norvig [26] provide an in-depth view of different aspects of AI.

ML first subdivides into:

— *Supervised learning*: Given a set of inputs/independent variables/predictors *x* and outputs/dependent variables/targets *y*, the goal is to learn a function *f*(*x*) that approximates *y*. This is accomplished by supervising *f*(*x*), that is, providing it with examples (*x*_1_, *y*_1_), …, (*x_n_*, *y_n_*) and feedback whenever it makes mistakes or accurate predictions.
— *Unsupervised learning*: Given several objects/samples *x*_1_, …, *x*_*n*_, the goal is to learn a hidden map *h*(*x*) that can uncover a hidden structure in the data. This hidden map can be used to ‘compress’ *x* (also known as dimensionality reduction) or to assign to every *x*_i_ a group *c*_k_ (also known as clustering or topic modelling).
— *Reinforcement learning*: Given an environment formed by several states ***s***_1_, ***s***_2_, …, ***s**_n_*, an agent, and a reward function, the goal is to learn a policy π that will guide an agent’s actions ***a***_1_, ***a***_2_, …, ***a**_k_* through the state space so as to maximize rewards.

**Figure 2.**
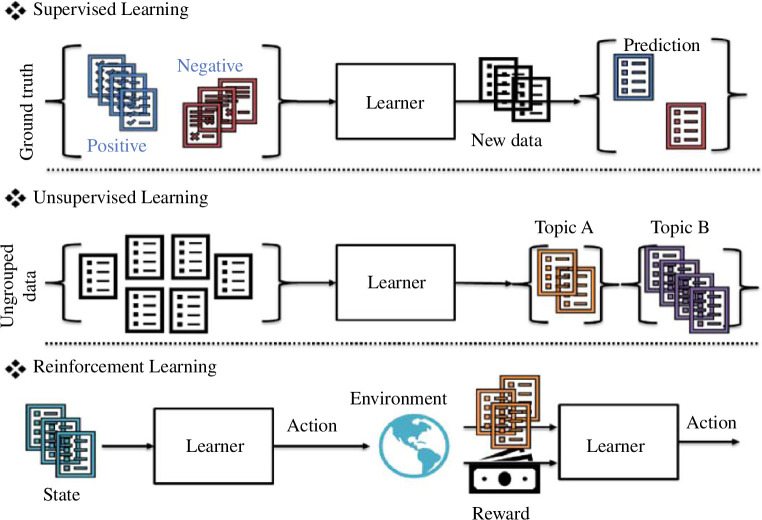
Main learning paradigms of machine learning.

Figure 2 provides an illustration of these key learning paradigms. Suppose a database of financial reports is available; if some of them have been historically labelled as positive and negative, we can leverage this to automatically tag future documents. This can be accomplished by training a learner in a *supervised* fashion. If these documents were unstructured, and spotting relations or topics is the goal (political events, economic data, etc.), a learner trained in an *unsupervised* manner can help uncover these hidden structures and relationships. Also, these documents can characterize the current state of the capital markets. Using that, a learner can decide which actions should be taken to maximize profits, hedge against certain risks, etc. By interacting and gaining feedback from the environment, the learner can reinforce some behaviours to avoid future losses or inaccurate decisions.

In addition to that, deep learning, adversarial learning, transfer and meta-learning are advanced new techniques enhancing supervised, unsupervised and reinforcement learning. They are not only powering new solutions and applications (e.g. driverless vehicles, smart speakers, etc.), but they are making the resolution of previous problems cheaper, faster and more scalable. They tend also to be more opaque, making the issue of auditing and assurance more challenging. The second subdivision is

**Figure 3.**
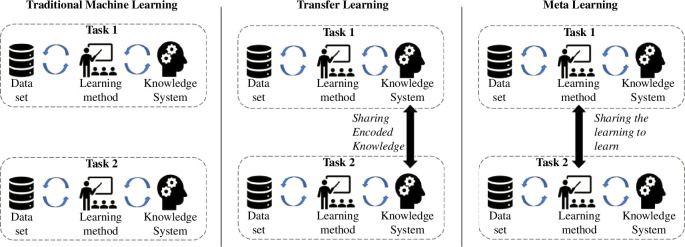
Traditional ML versus transfer learning versus meta-learning.

— *Deep learning*: deep learning algorithms attempt to model high-level abstractions in data by using multiple processing layers, with complex structures or otherwise, composed of multiple nonlinear transformations. Hence, the mapping function we are attempting to learn can be broken down into several compositional operations f(x)=f1∘f2∘f3∘ ⋯∘fn(x) . Various deep learning architectures such as deep neural networks, convolutional deep neural networks, deep belief networks and recurrent neural networks have been applied to fields like computer vision, automatic speech recognition, natural language processing, audio recognition and bioinformatics where they have been shown to produce state-of-the-art results on various tasks [27,28].
— *Adversarial learning*: adversarial machine learning is a technique employed in the field of machine learning that attempts to ‘fool’ models through malicious input. More formally, assume a given input *x* associated with a label ***c*** and a machine learning model *f* such that f(x)=c , that is, *f* can perfectly classify ***x***. We consider ***x*** ^∗^ an adversarial example if ***x*** ^∗^ is indistinguishable from *x* and f(x)≠c . Since they are automatically crafted, these adversarial examples tend to be misclassified more often than is true of examples that are perturbed by noise [29,30]. Adversarial examples can be introduced during the training of models, making them more robust to attacks from adversarial agents. Typical applications involve increasing robustness in neural networks, spam filtering, information security applications, etc. [31].
— *Transfer/meta-learning*: these two learning paradigms are tightly connected, as their main goal is to encapsulate knowledge learned across many tasks and transfer it to new, unseen ones. Knowledge transfer can help speed up training and prevent overfitting and can, therefore, improve the obtainable final performance. In transfer learning, knowledge is transferred from a trained model (or a set thereof) to a new model by encouraging the new model to have similar parameters. The trained model(s) from which knowledge is transferred is not trained with this transfer in mind, and hence the task it was trained on must be very general for it to encode useful knowledge with respect to other tasks. In meta-learning, the learning method (learning rule, initialization, architecture, etc.) is abstracted and shared across tasks, and meta-learned explicitly with transfer in mind, such that the learning method generalizes to an unseen task. Concretely, often in transfer learning a pre-trained model is moved to a new task [32,33], while in meta-learning a pre-trained optimizer is transferred across problems [34–36]. In both cases, the usual approach is to learn a deep neural network that can be reused later, usually by stripping some of its terminal layers and creating an encoder–decoder to match the input and output for a task. See figure 3 for a visual representation.

## 4. Main verticals of algorithm auditing

In computer science, there is a growing engineering expertise overlapping with the digital ethics space [37]. Issues of explainability, fairness, privacy, governance and robustness are now popular research themes among AI researchers—an area that falls under the umbrella of ‘trustworthy AI’ [1]. From an engineering point of view, we believe that the most mature and impactful criteria are:

— *Performance and robustness*: systems should be safe and secure, not vulnerable to tampering or compromising the data they are trained on.
— *Bias and discrimination*: systems should avoid unfair treatment of individuals or groups.
— *Interpretability and explainability*: systems should provide decisions or suggestions that are understandable by their users, developers and regulators.
— *Algorithm privacy*: systems should be trained following data minimization principles as well as adopt privacy-enhancing techniques to mitigate personal or critical data leakage.

The next subsections will deal with each one of these criteria.

### 4.1. Performance and robustness

Performance and robustness, as a technical concept, is closely linked to the principle of prevention of harm [38]. Systems should neither cause nor exacerbate harm or otherwise adversely affect human beings. This entails the protection of human dignity as well as mental and physical integrity, by ensuring that the automation of decisions and processes does not adversely impact human wellbeing and opportunities. For example, it has been argued that the automation of fairness is inherently unfair [39] and is not something that can be achieved under current laws, particularly in the EU, where fairness is judged on a case-by-case basis. Preventing harm can also entail consideration of the natural environment and the living world, as well as weighing up whether processes can be automated [40].

Most of the legal basis is established by the interaction between regulatory agencies, professional associations and industry trade groups, where standards, rules and codes of conduct are created:

— Finance: SEC, FCA, FSB, BBA and BIS
— Power systems: FERC and IEEE
— Electrical appliances: NIST, Nat Fire Protection Association and state legislation
— Automotive sector: National Transportation Safety Board and Soc Auto Engineers

*Algorithm performance* and *robustness* are characterized by how effectively an algorithm can be deemed as safe and secure, not vulnerable to tampering or compromising the data it is trained on. We can rate an algorithm’s performance and robustness using four key criteria [38]:

— *Resilience to attack and security*: AI systems, like all software systems, should be protected against vulnerabilities that can allow them to be exploited by adversaries, such as data poisoning, model leakage or the infrastructure, both software and hardware. This concept is linked with the mathematical concept of *adversarial robustness* [41], that is, how would the algorithm have performed in the worst-case scenario? Mathematically, this can be expressed as:
  Adversarial risk^1^: $E_{\left(x,\;y\right)∼p}\left[\underset{\delta \in \Delta \left(x\right)}{max}L\left(y;f\left(x+\delta \right)\right)\right]\approx \underset{\left(x,y\right)\in D^{val}}{mean}\left[\underset{\delta \in \Delta \left(x\right)}{max}L\left(y;f\left(x+\delta \right)\right)\right].$

— *Fallback plan and general safety*: AI systems (and the associated infrastructure) should have safeguards that enable a fallback plan in case of problems. Also, the level of safety measures required depends on the magnitude of the risk posed by an AI system. This notion is strongly associated with the technical concept of *formal verification* [42], which in broad terms means: does the algorithm attend the problem specifications and constraints? (e.g. respect physical laws). One way to express this mathematically is
  Verification bound^1^: $P\left(F\left(x;f\left(x\right)\right)\leq 0\right)\approx \;\frac{\#\left(F\left(x^{nom};f\left(x\right)\right)\leq 0\right)}{|S_{in}\left(x^{nom},\;\delta \right)|}.$

— *Accuracy*: pertains to an AI system’s ability to make correct judgements, for example, to correctly classify information into the proper categories, or its ability to make correct predictions, recommendations or decisions based on data or models. Accuracy as a general concept can be quantified by estimating the *expected generalization performance* [43], which means that in general the question of ‘*how well does the algorithm work?’* is asked (e.g. in 7 out of 10 cases, the algorithm makes the right decision). Typically, the expected generalization performance can be expressed by the following formula:
  Expected loss^1^: $E_{\left(x,\;y\right)∼p}\left[L\left(y;f\left(x\right)\right)\right]\approx \underset{\left(x,y\right)\in D^{val}}{mean}\left[L\left(y;f\left(x\right)\right)\right].$

— *Reliability and reproducibility*: a reliable AI system is one that works properly with a range of inputs and in a range of situations, while reproducibility describes whether an AI experiment exhibits the same behaviour when repeated under the same conditions. This idea is tied with the software engineering concept of *continuous integration* [44], that is, is the algorithm auditable? (e.g. reliably reproduce its decisions).

### 4.2. Fairness, bias and discrimination

Fairness as an ideal has been present in different manifestos and charters throughout history, gradually amplifying its outreach across the population, most notably in the UN Universal Declaration of Human Rights (1948). Most of the legal bases were developed after multiple public demonstrations, civil rights movements, etc. and are in many situations set or upheld at constitutional levels. We can mention a few across different countries. United States: Civil Rights Act (1957 and 1964), Americans with Disability Act (1990); United Kingdom: Equal Pay Act (1970), Sex Discrimination Act (1975), Race Relations Act (1976), Disability Discrimination Act (1995) and Equality Act (2010); and those enshrined in the constitutions of France, Germany, Brazil and many other countries. Indeed, it suffices to say that notions of fairness appeal to substantive value claims rooted in differing philosophical approaches and traditions—as such there are often ambiguous interpretations of the word ‘fairness’.

In AI and ML, there are multiple sources of bias that explain how an automated decision-making process becomes unfair, where the majority of these relate to the training data and are a particular problem for systems trained on real-world data [38]:

— *Systemic or historical biases*: ML systems reflect bias existing in the old data caused by human and societal biases (e.g. recruitment).
— *Feedback loops*: future observations confirm predictions made, which creates a perverse, or self-justifying feedback loop (e.g. police records).
— *Limited features*: features may be less informative or reliably collected for minority group(s).
— *Sample size disparity*: training data coming from the minority group are much less than those coming from the majority group.
— *Proxies*: even if protected attributes are not used for training a system, there can always be other proxies of the protected attribute (e.g. neighbourhoods).

To diagnose and mitigate bias in decision-making, we first need to differentiate between individual and group level fairness. (i) *Individual*: seeks for similar individuals to be treated similarly. (ii) *Group*: splits a population into groups defined by protected attributes and seeks for some measure to be equal across groups. There are multiple ways to translate these concepts mathematically [45–47]; and deciding which definition to use must be done in accordance with governance structures and on a case-by-case basis. Also, within group fairness, it is possible to distinguish between the aim of equality of opportunity and outcome. For example, using features extracted from a video interview to make recommendations about employability.

— *Equality of opportunity* worldview says that individuals are treated equally and given the same opportunities irrespective of their subgroup membership. A mathematical definition that is often used is the average odds difference [48]:

(4.1) $$AOD=\;\frac{1}{2}\;\left[\left(FPR_{group\;A}-FPR_{group\;B}\right)+\left(TPR_{group\;A}-TPR_{group\;B}\right)\right],$$

with FPR and TPR representing the false and true positive rates, respectively. The underscored groups A and B reflect the conditioning of FPR and TPR to a given subset of the population analysed (e.g. group A could represent young individuals and group B adult individuals).

— *Equality of outcome* worldview says that the extracted features should be related to ability rather than subgroup membership. In other words, scores across groups should be equal if ability across groups is equal. Statistical parity difference (SPD) [48] is generally the most adopted form to represent this idea symbolically:

(4.2) $$\begin{array}{l} SPD=\frac{P(\hat{y}=1\;|group\;A)}{P(\hat{y}=1\;|group\;B)}\approx \frac{Freq(\hat{y}=1\;|group\;A)}{Freq(\hat{y}=1\;|group\;B)}, \end{array}$$

with Freq representing the empirical frequency of positive/yes/etc. predictions *ŷ* made by the model.

We can also list variations of both, like equal reliability (UK-CDEI, 2021). Calibration is also capable of perpetuating pre-existing biases. It should be noticed that fairness could be interpreted radically differently in different environments and countries, and, hence, one deployment of a given algorithm may encounter several different fairness measurement barriers. Finally, it is perhaps worth noting that it is not mathematically possible to construct an algorithm that simultaneously satisfies all reasonable definitions of a ‘fair’ or ‘unbiased’ algorithm [45].

### 4.3. Interpretability and explainability

Being able to provide clear and meaningful explanations is crucial for building and maintaining users’ trust in automated decision-making systems [49]. This means that processes need to be transparent, the capabilities and purposes of systems openly communicated, and decisions—to the extent possible—explainable to those directly and indirectly affected. Without such information, a decision cannot be duly contested [38]. The ultimate user benefits from being able to contest decisions, seek redress and learn through user–system interaction; the developer also benefits from a transparent system by being able to ‘debug’ it, uncover unfair decisions and from knowledge discovery.

Hence, the capabilities and purpose of algorithms should be openly communicated, and decisions be easily explainable to those directly and indirectly affected. These must be done in a timely manner and adapted to the expertise of the stakeholder concerned (e.g. layperson, regulator or researcher). In the United States, credit scoring has a well-established right to explanation legislation via the Equal Credit Opportunity Act (1974). Credit agencies and data analysis firms such as FICO comply with this regulation by providing a list of reasons (generally, at most four per interpretation of regulations). From an AI standpoint, there are new regulations that give the system’s user the right to know why a certain automated decision was taken in a certain form—Right to an Explanation—EU General Data Protection Regulation (2016).

In the context of AI and ML, explainability and interpretability are often used interchangeably, although they are distinct [50]. *Algorithm interpretability* is about the extent to which a cause and effect can be observed within a system and the extent an observer is able to predict what will happen, for a given set of input or algorithm parameters. *Algorithm explainability* is the extent to which the internal mechanics of an ML (deep learning) system are explainable in human terms. In simple terms, interpretability is about understanding the algorithm mechanics (without necessarily knowing why); explainability is being able to explain what is happening in the algorithm.

**Figure 4.**
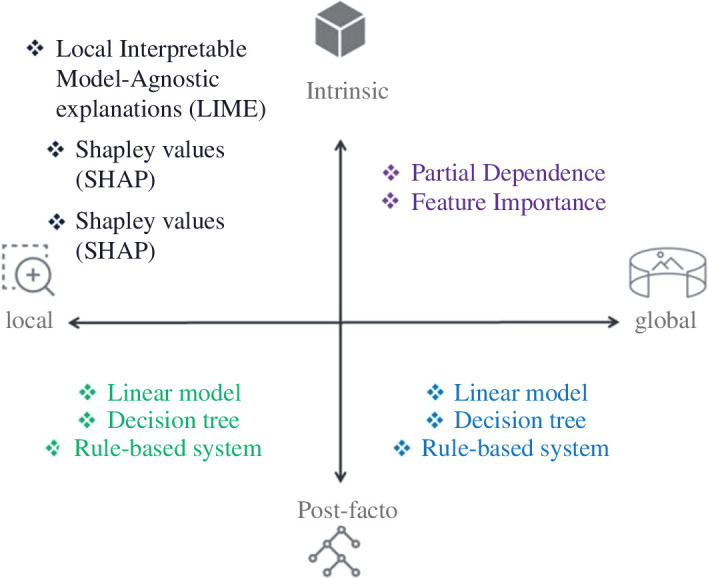
Types and levels of algorithm explainability.

There are multiple approaches to generate and provide explanations based on an algorithmic decision-making system. Figure 4 presents the types and levels of explainability: model-specific and agnostic, global and local [51,52]. Below, we unwrap these concepts, as well as outline some technical solutions.

*Intrinsic*: With intrinsic explainability, a model is designed and developed in such a way that it is fully transparent and explainable by design. In other words, an additional explainability technique is not required to be overlaid on the model in order to be able to fully explain its workings and outputs.

*Post-facto*: With post-facto explainability, a mathematical technique is applied to the outputs of any algorithm including very complex and opaque models in order to provide an interpretation of the decision drivers for those models.

*Global*: This facet focuses on understanding the algorithm’s behaviour at a high/dataset/populational level. The typical users are researchers and designers of algorithms since they tend to be more interested in the general insights and knowledge discovery that the model produces rather than specific individual cases.

*Local*: This facet focuses on understanding the algorithm’s behaviour at a low/subset/individual level. The typical users of local explanations are individuals being targeted by an algorithm, as well as members of the judiciary and regulators trying to make a case about potential discrimination.

It is important to note that the explainability requirements may be different for different regions and different use cases. This means that the same approach may not be applicable in all contexts of deployment of a given algorithm.

### 4.4. Algorithm privacy

From the principles level, privacy is closely linked to the principle of prevention of harm [38]; systems can cause or exacerbate adverse impacts owing to asymmetries of power or information, such as between employers and employees, businesses and consumers or governments and citizens. Preventing harm demands bespoke data governance that covers the quality and integrity of the data used, its relevance considering the domain in which the algorithm will be deployed, its access protocols, and the capability to process data in a manner that protects privacy. It is possible to group these issues in two key areas:

— *Privacy and data protection*: systems must guarantee privacy and data protection throughout a system’s entire lifecycle [53,54]. This includes the information initially provided by the user and the one generated about the user over the course of their interaction with the system. Finally, protocols governing data access should be put in place, outlining who can access data and under which circumstances [55].
— *Model inferences*: the security of any system is measured with respect to the adversarial goals and capabilities that it is designed to defend against. In this sense, one needs to provide information about (i) the level of access the attacker might have (‘black-box’ or ‘white-box’), (ii) where the attack might take place (inference or training) and (iii) passive versus active attacks [56].

Therefore, the risk assessment of algorithm privacy can be disentangled in ‘data’, ‘algorithm’ and the interaction between both components. Below, we outline the key methods available to assess risks coming from each of these elements.

**Figure 5.**
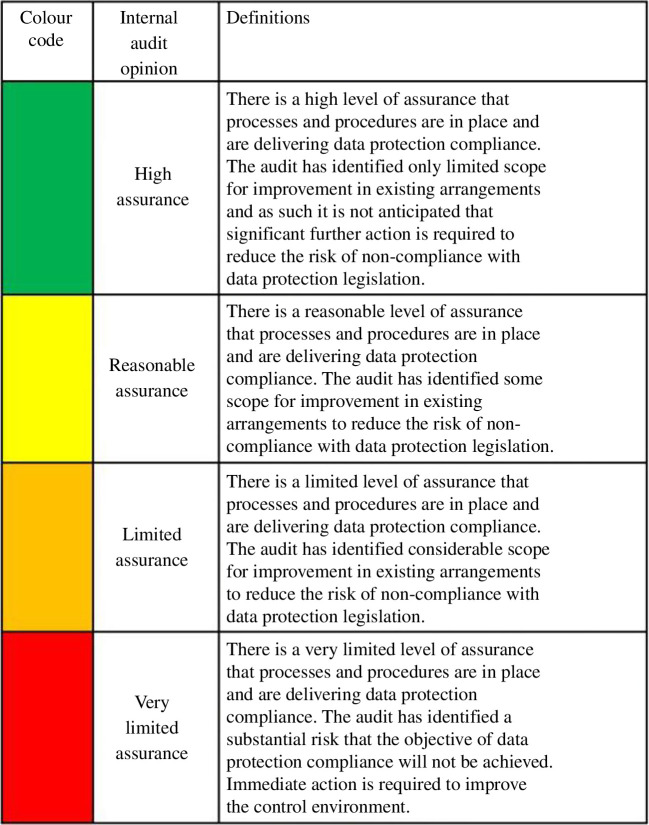
UK’s Information Commissioner’s Office has a colour-coded ‘Assurance Rating’ for data. Available at: https://ico.org.uk/for-organisations/audits/.

— *Data*: the standard procedure to assess risks in this vertical is the Data Protection Impact Assessment [57]. This procedure has been legally formalized in many jurisdictions, such as in the European Union, United Kingdom, Canada, California and Brazil. In the United Kingdom, as shown in figure 5, a qualitative rating can be provided depending on the perceived level of data protection. Another vector is data poisoning [58], where an attacker maliciously manipulates the training data in order to affect the algorithm’s behaviour.
— *Algorithm*: the key attack vector in this component is inferring model parameters and building ‘knock-off’ versions of it. To assess vulnerability, the auditor could apply techniques that aim to extract a (near-)equivalent copy or steal some functionalities of an algorithm [59–61].
— *Data–algorithm interaction*: the attack vectors in this component are inferring about members of the population or members of the training dataset through interactions with the algorithm. Attacks such as statistical disclosure [62], model inversion [63], inferring class representatives [64], membership and property inference [65–67] are different criteria that can be applied to an algorithm to assess levels of vulnerability.

### 4.5. Interactions and trade-off analysis

**Figure 6.**
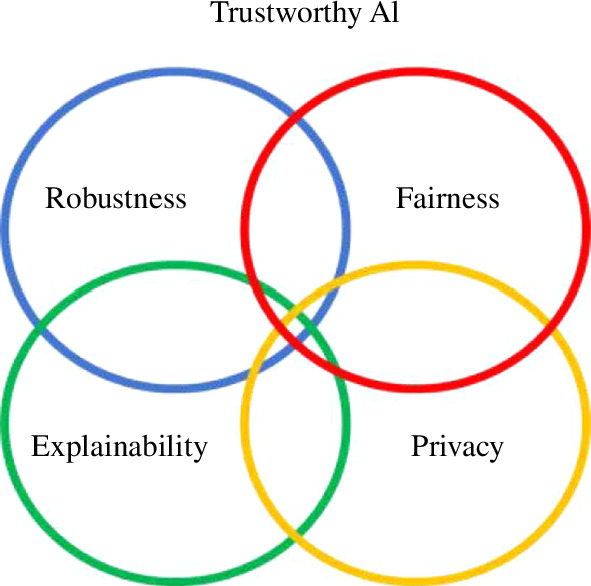
The overlaps between algorithm robustness, fairness, explainability and privacy.

As depicted by figure 6, risk verticals are not independent of each other—they overlap and interact. For example, debiasing procedures affect the model performance, global and local interpretation and, potentially, data minimization aspects. Having a clear understanding of what will be traded as a consequence of improvements in one vertical is becoming less of a technological concern and gradually more of a requirement across a wide array of guidelines [38,68,69]. Above all, it presents growing evidence that in the emerging area of trustworthy AI, hardly is there a solution, only trade-offs to be managed. Though the practicalities of trade-off analysis demand context, nonetheless some general explorations, roadmaps and guidelines can still be issued and performed. We explore some of these below.

**Figure 7.**
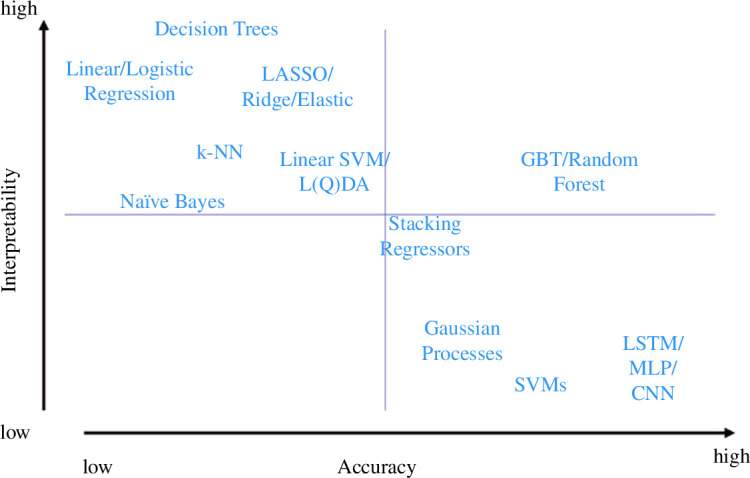
Algorithm selection trade-offs: model-specific interpretability versus accuracy.

*Explainability versus robustness (accuracy)*: one that has been extensively explored by different authors and organizations [69,70] is the *interpretability versus accuracy* trade-off—sometimes also presented as *explainability versus performance* trade-off. Figure 7 shows a typical depiction that can be found in many documents and papers. Prima facie, that is, looking only at the model function forms and training, the depiction is broadly accurate. However, such depiction is highly debatable in the light of data science practice since it could be that a linear model is the most accurate model, but owing to massive pre-processing performed (e.g. nonlinear features, etc.), the explainability level has been drastically reduced.

**Figure 8.**
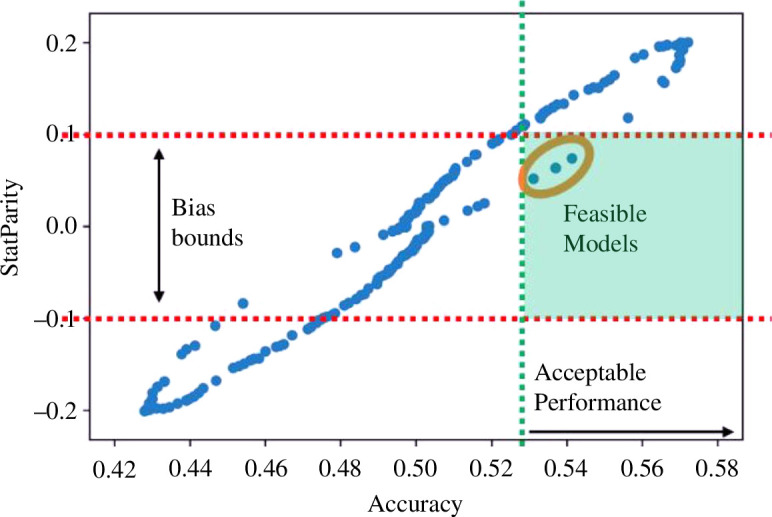
Algorithm selection trade-offs: bias (statistical parity) versus performance (accuracy).

*Fairness versus robustness*: another trade-off well explored in the literature is *fairness (in the form of algorithm bias) and robustness (in the form of algorithm performance*) [71,72,73]. Figure 8 explores a typical chart about this trade-off. Every dot represents an algorithm setup (parameters, hyperparameters, etc.); the work of an algorithm designer is to identify the acceptable *boundaries of statistical bias and performance*, for example, by adopting metrics like statistical parity and accuracy. These boundaries can be identified by liaising with business and end users, and by analysing best practices, standards or regulations commonly adopted in the field of application. In the example depicted in figure 8, the boundaries are set for −0.1 and 0.1 for bias (statistical parity), and the minimum acceptable performance of 0.53. From that, we can draw the region of algorithm configurations (or even models) that dwells within such limits. In this case, only three configurations are feasible from a fairness versus robustness point of view.

**Figure 9.**
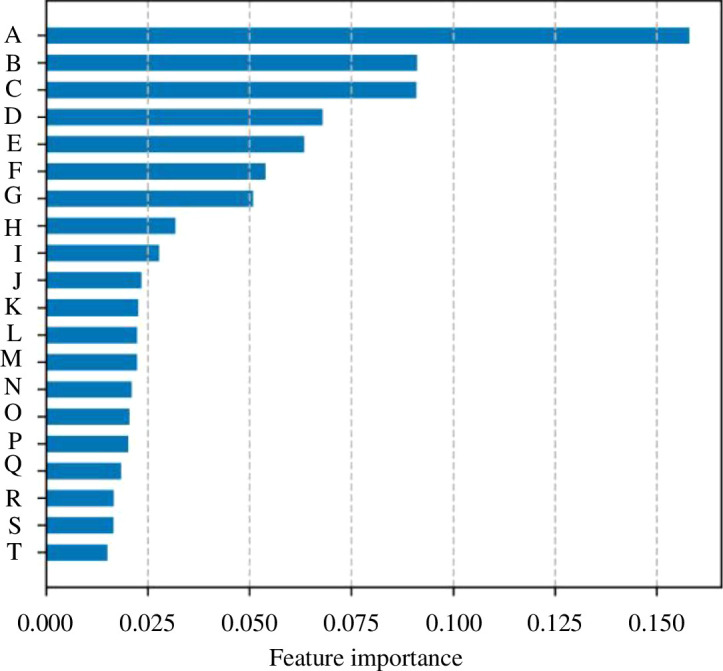
Algorithm selection trade-offs: explainability (feature importance) versus privacy (data minimization).

*Explainability versus privacy*: prima facie, the easier it is to interpret a model, the harder it is to conceal information or its judgement. Hence, at first sight, interpretability and privacy are negatively related. However, being able to explain a model’s internal workings such as via feature importance charts can aid with data minimization [74], a key pillar of algorithm privacy. Using figure 9 as an example, if we set a threshold of 0.025 to the feature importance metric, we can reduce the number of variables being used from 20 to only 8. Knocking-off variables ease the explanation of model judgements and will also reinforce to the end users that their information is used in an efficient manner but could leave the model or indeed data being more vulnerable to being reverse-engineered.

**Figure 10.**
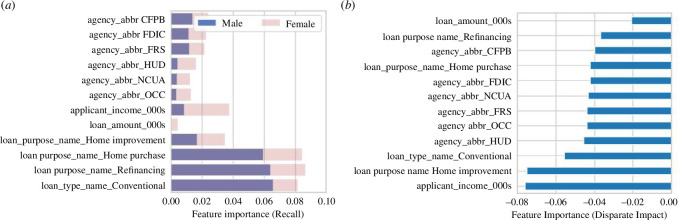
(*a*) Feature importance chart with the breakdown per male and female groups. (*b*) Explainability (feature importance) based on a bias (disparate impact) metric.

*Fairness versus explainability*: improving the explainability of a system as a means to achieve greater transparency of its use acts as a positive driver to uncover inherent bias and discrimination to all its users and designers (e.g. [75]). Figure 10*a*,*b* presents examples using feature importance charts to understand the key drivers for a mortgage application processing algorithm. Figure 10*a* demonstrates that when we break down the feature importance chart per declared sex, we discover disparities in how the algorithm is making its judgement—even though we have not included this information as an input to the model. Loan amount and particularly applicant income are significantly more relevant variables for female applicants than for male. We can perform a similar analysis, such as in figure 10*b*, where a permutation importance method was used on the disparate impact metric (male–female) to construct the feature importance chart. We uncover that there are disparities, as perceived in figure 10*a*, particularly with the loan purpose, type and applicant income.

**Figure 11.**
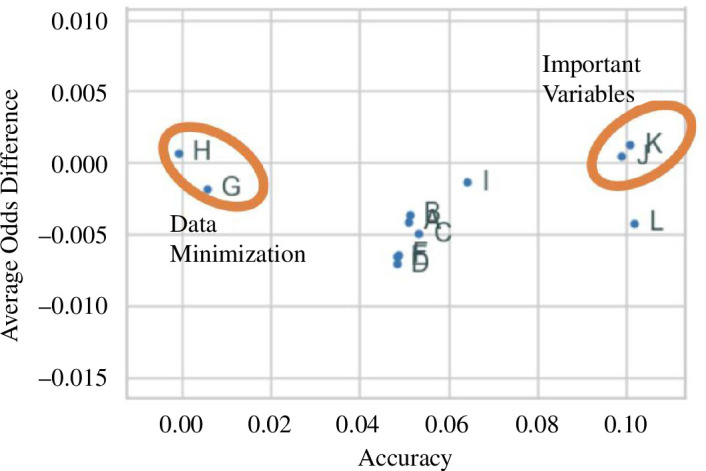
Interaction between all verticals. The values displayed were estimated by permutation importance using accuracy and average odds difference as loss metrics.

*Interaction between all verticals*: there are a few charts that can be crafted to display components of each vertical. Figure 11 displays one of such, where the key goal is to identify relevant variables and undertake data minimization. Relevant variables are defined as having a high impact on an algorithm performance (accuracy) and a low impact on an algorithmic bias (average odds difference)—both can be estimated by permutation importance using each as the loss metric. The variables J and K are key variables, meeting both criteria; the variables G and H could be eliminated since they do not affect much the model performance. Having this global understanding of an algorithm’s behaviour will become an unprecedented component to build and enhance the trustworthiness of an algorithm.

Two other interactions are worth briefly mentioning:

— *Robustness versus privacy*: both criteria are strongly connected, with techniques coming from the privacy literature like adversarial testing [56] percolating to robustness, and defence mechanisms built by the robustness [76] community looping back.
— *Privacy versus fairness*: respect for privacy and fairness within the same system introduces the question of trade-offs between the two values. From the perspective of privacy, particularly in cases of personal data, the further a system is to anonymity the more ‘private’ it can be said to be. Conversely, in the case of fairness, the concern is that systems perform equally for all protected attributes and, as such, systems need to be as transparent as possible for fairness to be assured. The tension between privacy and fairness becomes apparent, where a greater degree of privacy is likely to come at the price of fairness concerns [77].

Notwithstanding the critical nature of trade-off analysis, it should be noted that the intersection of all these areas is often impossible to achieve and not always desirable. Trade-offs should be seen as a way of finding an operational profile that is consistent with the needs of the application, rather than some abstract goal that needs to be achieved for a notion of ‘completeness’. We also note that while many of these trade-offs require a technical approach, achieving an equilibrium can be supported by the establishment and implementation of robust governance processes. For example, the explainability of a system can be supported by ensuring that there are comprehensive documentation practices to support the explainability of model development and specifications without affecting performance [78].

### 4.6. Future investigations

One of the key challenges is to define what risks should be prioritized and measured. This could be solved on a case-by-case basis; however, a roadmap or toolkit could be developed to provide business users and developers with the right recommendations and areas to focus on. In this perspective, future investigations could look at, given a specific algorithm, how to:

— Define the appropriate vertical or risks that should be prioritized as well as the right control levels for them.
  - *Bias and discrimination*, such as when the algorithm will affect individuals or groups.
  - *Performance and robustness*, such as when the algorithm can cause financial and reputational damage by not being statistically accurate or brittle.
  - *Interpretability and explainability*, such as when the lack of understanding of the decisions being made, suggestions being provided, or recourse is needed.
  - *Privacy*, such as when the possibility of leakage of intellectual property or private information is a feasible event.
— Monitor metrics and recommend interventions depending on the phase, information provided and the type of project involved.
  - *Development/procurement phase*: provide recommendations of useful tools and techniques to include so that risks can be mitigated and avoided.
  - *Deployment phase*: request information about performance, bias and other metrics that are needed to assure that the risks are under control.

## 5. Levels of access for auditing

As previously discussed, the level of access that an auditor has during an investigation of an algorithm can vary. While the common practice in scientific literature and technical reports is to categorize the knowledge about the system in two extremes, ‘white-box’ and ‘black-box’, we contest that the spectrum about the knowledge of a system is more of ‘shades of grey’, that is, a continuum, than this simple dichotomy. This additional nuance allows a richer exploration of the technologies available for assessment and mitigation, as well as the right level of data disclosure that a certain business feels comfortable to engage.

Hence, we can identify seven levels of access that an auditor can have to a system (table 2). It ranges from ‘process-access’ where only indirect observation of a system can be made to ‘white-box’ where all the details encompassing the model are disclosed. The levels in between are set by limiting access to the components behind the learning process (e.g. knowledge of the objective function, model architecture, training data, etc.).

This categorization has the following two monotonic properties:

— *Detail*: accuracy and richness increase with levels.
— *Concealment*: information concealed decreases with levels.

**Figure 12.**
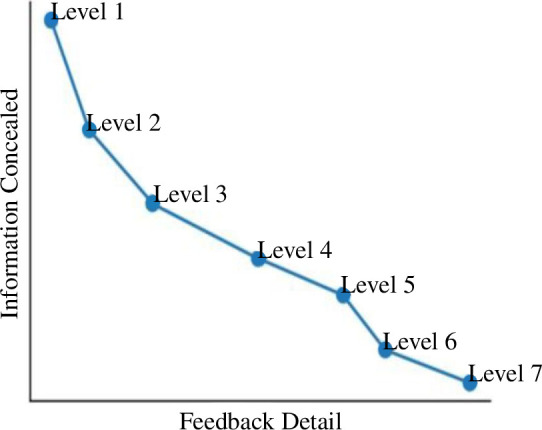
Information concealed versus feedback detail trade-off curve.

In what follows, we explore the trade-off: detail and concealment (figure 12). It is worth mentioning that Level 7 access allows all the analysis of the above levels, simply because we have full access to the algorithm. Conversely, analysis and techniques requiring Level 7 cannot be used at Level 6 without proper assumptions. Hence, Level 7 contains all the assessment, monitoring and mitigation strategies of upper levels, with the report getting less detailed and inaccurate as levels increase.

### 5.1. Level 7: ‘white-box’ auditing

In the ‘white-box’ setup, the auditor knows all the details encompassing the model: architecture or type *f*, learning procedure and task objectives *L*, parameters *θ*, output *y* and input *x* data used to train and validate the model and the access to perform predictions *f*(.).

This level of access, identical to the access that the system developer and business user have, allows the auditor to provide accurate and richer feedback. Accurate because the whole assessment can be performed using the actual system and based on fewer or no assumptions, and richer because the number of tests and recommendations that can be made range from the actual model selection to training, bias mitigation, validation and security. It would be easier to assess mitigation strategies and provide actual information that can be more easily documented by the developers.

This level of access is more appropriate for internal auditors or in-house consultants since this would demand an additional level of disclosure that may require non-disclosure, intellectual property sharing, data sharing, etc. agreements in place.

### 5.2. Level 6: learning goal

In the learning goal setup, the auditor knows most of the details encompassing the creation and purpose of the predictive system: learning procedure and task objectives *L*, parameters *θ*, output *y* and input *x* data used to train and validate the model and the access to perform predictions *f*(.).

From a modelling point of view, the auditor knows how to refit/re-learn the model using the actual incentives/objective function that it was trained on *L*(*f*_θ_(*x*),*y*), but without knowing the model *f* is family (e.g. kernel method) or components (e.g. number of neurons).

This level of access allows the auditor to investigate an almost accurate picture of the system, without necessarily infringing on much of the intellectual property. The feedback has a high degree of detail, with information on the model complexity, stress-testing and trade-off analysis of bias, privacy and loss being able to be performed without little to no assumptions. This level of access is enough to perform automated internal and external auditing since the human involvement after setting up the APIs and environments is considerably low.

### 5.3. Level 5: parameter manipulation

In the parameter manipulation setup, the auditor can recalibrate/reparametrize the model but has no information on its type or family, and what is the incentives/objective function it was built on. Hence, the auditor has access to parameters *θ*, output *y* and input *x* data used to train and validate the model, and the access to perform predictions (.).

This level explicitly allows the auditor to perform stability and perturbation analysis on the model *f_θ_*. Hence, it can provide reasonable feedback, particularly covering areas of how stable the system is performing, its judgements and the explanations being provided. Also, it would allow the auditor to assess the risk of functionality stealing from a privacy point of view. This level of access is relatively straightforward to implement via an API and can be easily automated for external auditing. The level of information known about the model nature is relatively low, allowing low infringement of intellectual property or disclosures of another nature. In addition, since the auditor can reparametrize the model, and based on certain assumptions, the auditor can in practice retrain the model.

### 5.4. Level 4: outcome access (‘grey-box’)

In the outcome access level, the auditor has the capacity to make predictive calls with the model using the actual training data and to compare it with outcome/output/target information. Therefore, the auditor has access to output *y* and input *x* data used to train and validate the model and the access to perform predictions (.).

This setup is deemed by some authors as ‘black-box’ since the auditor does not know the parameters and architecture of the model. From a modelling perspective, a host of techniques are available to assess and operate at this level, most of them under the umbrella of ‘model-agnostic’ procedures (e.g. cross-validation, Shapley values, etc.).

Since there are higher levels of non-access, we deem this level as ‘grey-box’ since some information is still known to the auditor. With the available access and based on a few assumptions, the auditor can perform concept drift analysis, investigate the accuracy of explanations, perform inversion attacks and check bias from an equality of opportunity point of view (e.g. equal odds difference). The auditor can also build baseline or competitor models to *f*.

Depending on the specifics, this yields a high to medium level of detail in the final feedback provided. From this level onwards, apart from data-sharing agreements, there is a little to no need to share intellectual property or development details. The level of automation that can be achieved and implemented makes it possible to perform most analyses quicker and possibly in real time.

### 5.5. Level 3: training data access

In the training data access setup, the auditor has the capacity to make predictive calls with the model using the actual data that has been used to train and validate it but cannot compare the predictions with the actual data. That is, the auditor has only access to input *x* data used to train and validate the model and the access to perform predictions *f*(.).

The absence of outcome information *y* makes the problem of assessing the generalization behaviour of a model hard, particularly to assess its performance. Since only the predictions *f*(*x*) are available, some analysis can still be performed, like computing bias from an equality of outcome perspective (e.g. disparate impact), property and membership inference or creating surrogate explanations. Synthetic data, near the actual distribution of the input *x*, can be generated, allowing for an investigation of the model’s brittleness to gradual changes in the distribution.

### 5.6. Level 2: model access (‘black-box’)

In the model access level, the auditor has the possibility to make predictive calls with the model but without having any information about the actual distributions of the training data. Some metadata could be shared, for example, the name of the variables, types, ranges, etc. Therefore, the auditor has only access to perform calls in *f*(.) using some artificial input *x* ^∗^.

This level of access entails the least amount of information disclosed to the auditor since no data-sharing agreements are needed. The level of automation that can be achieved is very high since only API access is needed to perform the analysis. Most of the quantitative analysis performed is centred around an adversary setup, resembling the work of threat models performed in the privacy space. Adversarial attacks, adversarial evaluation of bias and discrimination (fairness), extracting feature relevance and partial dependency explanations and different forms of privacy attacks (under the umbrella of statistical disclosure) are typical analyses that could be performed.

### 5.7. Level 1: process access

In the process access setup, the auditor has no direct access to the algorithm, with its investigations and interventions occurring during the model development process. With the impossibility of performing calls at the model *f*, the auditor depends on checklists that can be partially qualitative and quantitative information. General and sector-specific guidelines issued by regulators and other governmental bodies supplemented by a combination of company/application-specific could form the body of the assessment. Probably for low-stakes and low-risk applications, this level of disclosure and feedback detail might be the most appropriate.

We believe that the above level of access scheme can be used by regulators and standard bodies in the context of balancing proprietary respect and risk, where context and sector sensitivities will be critical in deciding the level of access required.

#### 5.7.1. Future investigations

One of the key challenges is to specify which types of processes would be in play at each of these levels. For example, for each level, how much interaction would the auditor need with the company being audited? One can imagine that for the deepest level of auditing, it may be necessary to first interview the key people in the company to ascertain their desires and goals for the operational parameters of the algorithms. Conversely, for the lowest level of auditing, simple checklists and self-assessment forms may be sufficient. Perhaps also, automated tooling running over data and algorithms to produce high-level analysis.

On a more methodological dimension, it is difficult for those with limited technical knowledge, such as a non-technical executive or regulator, to assess which is the right level of auditing/oversight needed for a given algorithm. A roadmap or toolkit could be employed to set the right level of oversight needed for the AI application being developed or acquired:

— ‘Checklist level’: when the risks are low, and no oversight is needed.
— ‘Black-box level’: when the risks are low-medium and little oversight is needed.
— ‘Grey-box level’: when the risks are medium and some oversight is needed.
— ‘White-box level’: when the risks are medium to high and full oversight is necessary.

To this end, there are emerging legislative requirements for algorithm audits, including the Digital Services Act (DSA) in the European Union and Local Law 144 in New York City. Although implicit, each law sets minimum requirements for the level of access that auditees must grant auditors, which auditees can choose to go beyond for a more comprehensive audit. Indeed, these two laws take very different approaches to algorithm auditing, with the DSA requiring only process access (Level 1), where auditors are required to assess the risk mitigation processes put in place by online platforms, while Local Law 144 requires outcome access only (Level 4) to measure subgroup differences in outcomes. Codifying audits in legislation in this manner ensures that the level of access is not left to the discretion of those without the deep technical knowledge required to conduct an audit, maximizing the value of the audit and legal compliance.

## 6. Mitigation strategies

**Figure 13.**
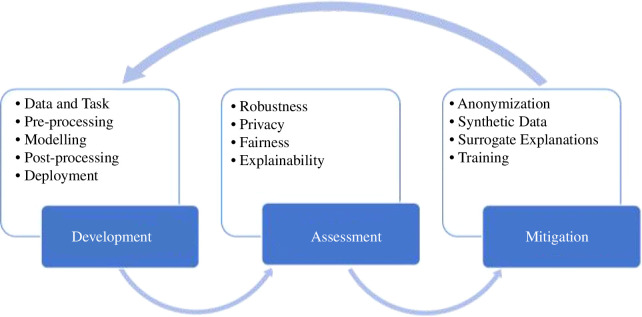
Feedback loop: from model development, assessment and mitigation, to redevelopment, reassessment and re-mitigation.

Mitigation strategies are a set of techniques employed to address issues highlighted in the assessment part of algorithm auditing. They consist of specific procedures that can be used in conjunction to enhance an algorithm’s performance and solve issues like algorithm debiasing or establishing surrogate explanations. To some extent, they act as ‘add-ons’ to certain stages of model development, and hence demand retraining and reassessment of the model—figure 13 establishes this feedback loop. We can highlight two types of mitigation procedures:

— *Human*: all procedures that involve how algorithm developers design, collaborate, reflect and develop algorithms. These procedures can involve (re)training, impact assessment, etc.
— *Algorithm*: all methodologies that can be applied to improve an algorithm’s current outcome.

These approaches are not in conflict and one solution may end up using both procedures in concert. In this section, we explore mainly the mitigation strategies that can be employed to improve an algorithm’s robustness, explainability, privacy and fairness.

*Performance and robustness*: each technical criterion listed in §4.1 embodies several technical mitigation strategies (table 4). These technical strategies can aid the analyst in measuring the expected generalization performance, detecting concept drifts, avoiding adversarial attacks and having best practices in terms of systems development and algorithm deployment.

**Table 4.** Mapping technical criteria and solutions for algorithm robustness and performance.

criterion	technical solution
expected generalization performance	cross-validation [43]: k-fold-cv, leave-one-out, etc. covariance-penalty [20]: Mallow’s Cp, Stein unbiased risk estimator concept drift [79,80]: gradual mitigation, abrupt correction and pre-emptive detection
adversarial robustness	evasion attacks: fast gradient sign method [81], DeepFool [82], etc. defence: label smoothing [76], variance minimization [83], thermometer encoding [84], etc.
formal verification	complete: satisfiability modulo theory [85,86], mixed integer programming [87], etc. incomplete: propagating bounds [88], Lagrangian relaxation [89], etc.
reliability and reproducibility	code versioning: Git (Github), Mercurial (BitBucket), etc. reproducible analysis: Binder, Docker, etc. automated testing: Travis CI, Scrutinizer CI, etc.

*Explainability and interpretability*: most interpretability and explainability enhancing strategies concentrate on processing and post-processing stages (table 5). We can split the procedures mainly into the model-specific and model-agnostic axis, with all model-specific approaches being able to provide global and local explanations by design (in-processing). Model-agnostic procedures act as a *post hoc* ‘wrapper’ around an algorithm, with some techniques only focusing on local explanations (e.g. LIME) or global explanations (e.g. partial dependency plots). The mitigation strategies need to consider the use case domain and level of risk, the organization’s risk appetite, all applicable regulations and laws and values/ethical considerations.

**Table 5.** Modelling stage and different technical solutions for algorithm explainability and interpretability.

stage/method	technical solution
in-processing/model-specific	rule-based explanations: decision trees, rule-induction methods model’s coefficients: linear regression, linear discriminant analysis nearest prototype: k-nearest-neighbour, naive Bayes
post-processing/model-agnostic	surrogate explanations: LIME [90], explainable boosting machines [91], PIRL [92] perturbation: gradient-based attribution methods [93], permutation Importance [94], SHAP [95] simulation analysis (what-if?): counterfactual explanations and algorithmic recourse [96,97]

*Bias and discrimination*: regardless of the measure used, algorithm bias can be mitigated at different points in a modelling pipeline: pre-processing, in-processing and post-processing [48]. Table 6 presents a snapshot of different methodologies to mitigate bias in AI systems.

**Table 6.** Modelling stage and different technical solutions for algorithm bias and discrimination.

stage	technical solution
pre-processing	reweighing subjects [98] oversampling minority groups [99] disparate impact remover [72] learning fair representations [100]
in-processing	adversarial debiasing [101] fairness constraint [73,102] counterfactual fairness [103]
post-processing	calibrated equality of odds [104] reject option classification [98]

*Algorithm privacy*: from an engineering standpoint, there are emerging privacy-enhancing techniques to mitigate personal or critical data leakage. These techniques can act in different moments of the system development: (i) during the pre-processing stage by feature selection, dataset pseudo-anonymization and perturbation; (ii) during in-processing by using federated learning, differential privacy and model inversion mitigation; and (iii) deployment by implement rate-limiting and user’s queries management. Table 7 presents these methods and key references.

**Table 7.** Modelling pipeline and different technical solutions for algorithm privacy.

stage	technical solution
pre-processing	data minimization by dim reduction [74] dataset (pseudo-)anonymization [105] dataset perturbation [106]
in-processing	federated learning [107,108] differential privacy [109,110] model inversion mitigation [63] data poisoning defence [111]
deployment	rate-limiting user’s query management

### 6.1. Future investigations

On the mitigation point generally, one assumes that the auditor would recommend the mitigation procedures that would need to be applied in order to address identified issues. Perhaps they would recommend a range of options or require a given mitigation mechanism to be performed. Different levels would demand different timelines and activities. Figure 13 fleshes out the general perspective, but one could explore in more detail what could be done on different levels, such as

— Level 7: ‘white-box’ level
  - starts with an interview for goals and context with the development and business team;
  - deep dive to examine the system with the development team;
  - write a report with the details of the system and the business problem it is aiming to solve as well as recommendations to improve it;
  - mitigation strategies are implemented, and the system is re-developed;
  - another audit is performed to assure that the key performance metrics are attained.
— Level 1: ‘checklist’ level
  - starts with a self-assessment performed by the team developing the system;
  - depending on the stage of development and verticals to be prioritized, recommendations of interventions or metrics are reported;
  - a final documentation is issued with possible monitoring and checkpoints for further assessment.

## 7. Assurance processes

The broader outcome of an auditing process is to improve confidence or ensure trust in the underlying system. After assessing the system and implementing mitigation strategies, the auditing process assesses whether the system conforms to regulatory, governance and ethical standards. However, it should be noted that this declaration does not necessarily mean that the system is compliant with other relevant laws depending on whether they were included in the framework used to inform the audit. Indeed, the focus of the audit could be compliance with a particular standard or code without taking into consideration other wider laws.

Providing assurance, therefore, needs to be understood through different dimensions and steps need to be taken so that the algorithm can be shown to be trustworthy.

**Figure 14.**
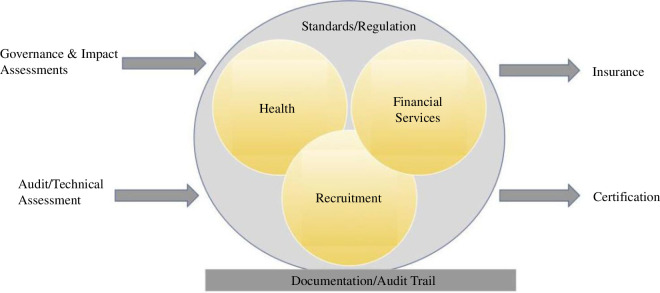
Diagram outlining the steps towards assurance: combining governance and impact assessments with audit and technical assessment; finding equivalent standards and regulations in the sector/end-application; generating a document/audit trail that will feed into certification and insurance as part of assurance.

Figure 14 outlines the steps towards assurance: combining governance and impact assessments with audit and technical assessment; finding equivalent standards and regulations in the sector/end-application; generating a document/audit trail that will feed into certification and insurance as part of assurance. We expand each point in the forthcoming subsections.

### 7.1. General and sector-specific

The satisfaction of a particular standard—e.g. certification, auditability, etc.—will become mandatory. We read this from the growing calls for AI, ML and associated algorithms to be responsibly developed and appropriately governed [13,68,112]. We anticipate that standards will be both general and sector-specific:

— *General standards*: the guidance (which may or may not be legally codified) will encompass broad dimensions such as privacy, explainability, safety and fairness, and these will be set by institutions and bodies with non-sector specific remits (e.g. the UK’s Information Commissioner’s Office). Developments in this space are becoming more concrete. For instance, in the publication of ‘Explaining Decisions Made with AI’, the Information Commissioner’s Office and The Alan Turing Institute [69] advise on how organizations can explain the processes, services and decisions delivered or assisted by AI to those that are affected by such decisions—the guidance outlines explanations in terms of who is responsible, data choices and management, fairness considerations, safety and impact. The International Organization for Standardization (ISO) and the International Electrotechnical Commission (IEC) have made the Artificial Intelligence Standard ISO/IEC 22989, which provides guidance on technologies such as natural language processing and computer vision and provides a comprehensive list of definitions to promote the development of a shared vocabulary freely available to the public. Furthermore, EU standards bodies CEN-CENELEC have been tasked with the development of standards to support the implementation of the EU AI Act over the next few years.
— *Sector standards*: sector-specific guidance already exists, which addresses idiosyncrasies of application. For example, the UK’s Financial Conduct Authority is leading in the debate on standardizing AI systems in financial services [113], the UK’s Care Quality Commission in ML development for medical diagnostic services [114], USA’s Department of Defence in the defence space [115]. In addition to sector-specific regulators issuing guidance, sectors themselves are developing their own standards and approaches to best practice. Recruitment is an example of this [116]. Application-specific standards, like the USA’s NIST for Facial Recognition [117], are a promising avenue.

### 7.2. Governance

Governance can be divided into two broad streams, namely technical and non-technical:

— *Non-technical governance*: concerns systems and processes that focus on allocating decision makers, providing appropriate training and education, keeping the human-in-the-loop and conducting social and environmental impact assessments. The issue of accountability and sector-specific particularities dominate the current debate; here, what is being referred to is
  - who will be liable if something goes wrong (processor, controller and user), that is, the allocation of responsibility;
  - what current legislation like GDPR, financial regulations, etc. have to say on a case-by-case basis; and
  - differences between countries and economic blocks.
    Within this context, there is also a literature on algorithmic impact assessments, which calls for doing a data protection impact assessment when algorithms are used [118–121]. Additionally, there are calls for AI impact assessments that address issues of human rights and social and environmental concerns (EU-HLEG, 2018) [122].
— *Technical governance*: concerns systems and processes that render the activity of the technology itself accountable and transparent. This touches upon ethical-by-design and technical auditing (involving the creation of quantitative metrics for tracing and tracking decisions, making the technologies accessible for verification and accountability). The main dimensions of technical auditing that will be explored are given in [13]:
  - *Robustness and performance*: systems should be safe and secure, not vulnerable to tampering or compromising—including the data they are trained on. Key concepts in this dimension are resilience to attack and security, fallback plan and general safety, accuracy/performance and reliability and reproducibility.
  - *Bias and discrimination* (fairness): systems should use training data and models that account for bias in data, to avoid unfair treatment of certain groups. By bias we mean, for example, yielding more false positives to a group in relation to another (young people versus older people, etc.). Key sources of bias include tainted or skewed examples, limited features, sample size disparity and proxies to protected attributes.
  - *Explainability and interpretability*: systems should provide decisions or suggestions that can be understood by their users and developers. Key techniques in this space are individual/local explanations, population/global explanations, model-agnostic and model-specific interpretations.
  - *Privacy*: systems should be trained following data minimization principles as well as adopt privacy-enhancing techniques to mitigate personal or critical data leakage. Key concepts in this area are data protection, quality, accuracy, integrity and access to data and decisions.

### 7.3. Monitoring interfaces

A risk-based approach, as observed in the European Commission’s white paper on AI and the German Data Ethics Commission [123], outlines two distinct notions of risks:

— *Sectoral*: where high risk is identified with respect to things such as healthcare, transport, energy and parts of the public sector (e.g. asylum, social security and employment services).

We note that all these sectors have the commonality of human impact, that is, whether a service, instruction, decision, etc. impacts a human user and citizen. This is a broad, abstract and blanketed approach, that is highly likely to result in two things: (i) *risk aversion* and (ii) *autonomous systems becoming a high-cost venture*. For example, a simple healthcare booking chatbot can become economically unfeasible because it falls under healthcare. Similarly, in the context of high-risk high-reward, a risk-based approach based upon sector will *discourage potentially high-positive impact algorithmic systems* (e.g. medical applications of AI have significant risk and lifesaving potential). As such, we believe this will stifle innovation.

— *Use*: the second notion of risk introduced is that ‘where use means that significant risk is likely to arise (risk of injury, death or significant material or immaterial damage)’.

A concern with this categorization of risk is that it is unclear how unintended consequences can be assessed. We argue that risk can be thought of in terms of known and unknown risks and technical and non-technical risks (presented in ‘risk matrix’ table 8) [124].

**Table 8.** Risk matrix outlining concerns and mitigation between technical/non-technical dimensions and known/unknown risks.

audit and impact assessment	known	unknown
technical	bias/fairness; safety; explainable; accessibility; data protection; trails; verification; comprehensibility	breakdown/robustness; nature of hack (theft; DOS)
non-technical	governance; oversight; whistle blowing; lack of education (education/training); authorization	trust; reputational; psychological and social impact; loss of skills

Given the problems referred to above and the vagueness of ‘risk’ in these calls, drawing from industry precedence, intuitive performance dashboard stop-light interfaces have been proposed. These will facilitate monitoring of performance over time [1,125], with green, amber and red representing high performance, satisfactory performance and poor performance, respectively. Furthermore, from a regulatory and standards standpoint, the UK’s Information Commissioner’s Office has a colour-coded ‘Assurance Rating’ for data (figure 5). A stop-light system can be used in several ways, like in the deployment phase where green, amber and red can be read in terms of how a system is performing in accordance with the purpose of its deployment. Within the context of assurance and audit the respective colours can be read in terms of high-performing/compliant (green), low-performing/compliant (amber) and non-compliant (red).

### 7.4. Unknown risks

Foundational to safety is that steps should be taken and procedures in place that *prevent harm*. This preventative approach requires that risks are anticipated in order to ensure that the chances of them occurring are mitigated, and if they do occur, then the impact is minimal. In order to do this, risk assessments are performed. In the context of the above, we can think of two kinds of risk assessment:

— *Technical audits*: conducted in the development phase and for live monitoring.
— *Impact assessments*: conducted before deployment and to design mitigation strategies.

Note that in table 8, the known technical and non-technical risks are covered by audit and impact assessment; this leaves unknown technical and non-technical risks, and one approach to address these is through ‘red teaming’ algorithms [1]:

— *Red teaming*: a systematic attempt to probe, expose flaws and weaknesses in a system, process, organization etc., both technical and non-technical, is undertaken. The ‘red team exercise’ assumes the persona of a hostile agent, with the hope that in exposing thereunto unanticipated weaknesses, that is, unknown risks, the risk mitigation can be improved.

Although there will still be unknown risks, it is hoped that best practices can be established through such activities; notwithstanding proprietary issues, this can be facilitated through knowledge transfer (via publication of methods to probe ‘attack’ and mitigate) [1].

### 7.5. Certification

Certification is part of the assurance process that confirms that a system, process, organization, etc. satisfies a particular standard. It is typically intertwined with regulatory requirements. However, certification can also be granted by industry bodies or other recognized authorities. We read certification as a final ‘stamp’ or confirmation, which can be achieved by providing evidence and proving that a system, process and organization have satisfied a given set of standards. Certification may come in a number of forms, including:

— *Certification of a system*: here, likely in line with national regulatory and standard bodies, the use of AI, that is, the systems and governance, may be certified as trustworthy or responsible. This may be akin to the granting of an organizational licence.
— *Sector-specific certification*: here, it is possible that sector standard bodies and regulators issue their own sector-specific certification.
— *Certification of a responsible agent*: good practice and industry standards within the context of data protection have led to the position of a ‘data protection officer’, and, by analogy, something akin to a ‘responsible AI Officer’ may emerge. These officers may be certified.
— *Certification of algorithm engineers*: here, the AI engineers may be certified, for example, by being granted a license by or admission into an accreditation organization (cf. trade association).

Another possibility is that certification may be issued for specific aspects of a system; here certifications for *robustness, explainability, privacy and bias and discrimination* may be issued.

### 7.6. Insurance

Closely related to assurance is the insurance of algorithms. It is possible that this will become a significant risk mitigation requirement for companies engaged in automation and as such a significant market for insurers. We envision that this will align closely with explainability and algorithm auditing in accordance with regulations and standards. Pricing such contracts will demand an understanding of the risks involved in each vertical of the algorithm system (robustness, bias, etc.) as well as indemnity insurance for high-risk sectors or high-risk end applications.

### 7.7. Future investigations

*Certification* is a topic that demands a section of its own. Questions related to: should one certificate be issued for the whole process or parts of the system? What could be shared with third parties to declare that the algorithms have been audited and verified? This brings us to the area of certificating authorities—who they are and what are their roles? How do they (if at all) differ from the auditor?

*Accountability roles* are a topic that also demands another section, separating the obligations of each of the players in the supply chain—the one that commissions the algorithm, the designer, the coder, the tester, the operator and so on. One can use analogies such as a comparison with the general product safety regulations, where the obligations are primarily on the manufacturer of goods, but the distributor and retailers have lesser but serious obligations to ensure safety.

## 8. Final remarks

This work is a first step towards understanding the key components underlying algorithm auditing. We provide a list of definitions and a taxonomy since this area is a combination of research done mostly in silos, such as bias and discrimination, robustness, explainability and privacy. Translating concepts such as accountability, fairness and transparency into engineering practice is non-trivial, with its impact perceived in design choices, algorithms to be used, delivery mechanisms and built infrastructure. This demands a full integration with respect to governance structures with real-time algorithm auditing.

We foresee that a new industry is emerging, Auditing and Assurance of Data and Algorithms, with the remit to professionalize and industrialize AI, ML and associated algorithms. Since the magnitude of the challenge will increase year-on-year for the foreseeable future, this industry will increasingly demand human capital (AI/digital ethicists and data scientists), RegTech-inspired solutions and business models [126] and (thought-)leadership from concerned regulators, politicians, NGOs and academics.

Below, we highlight related questions (which have not been covered extensively in this article):

— *AI, ML and algorithm ethics*: with the proliferation of AI research and deployment, along with high-profile cases of harm, awareness of the social impact and ethical implications of AI has risen to the fore. What is now referred to as ‘AI ethics’ or ‘trustworthy AI’ or ‘responsible AI’ is the body of literature that has resulted because of this consciousness and debate [127]. The field of AI ethics has undergone three broad phases [128]: principles, ethical-by-design approach, and indeed the current phase, which is concerned with the need to standardize and operationalize the AI ethics discipline.
— *Legal status of algorithms*: there is a growing discussion regarding algorithms and the law, in particular, concerns regarding fairness and automation [40] in the judiciary concerning the ‘status of algorithms in law’. In law, as we know, companies have the rights and obligations of a person. Algorithms are rapidly emerging as artificial persons: a legal entity that is not a human being but for certain purposes is legally considered to be a natural person [2]. The argument is that since algorithms are doing or intermediating business (agency) with humans, companies and even other algorithms they also need to have the status of an artificial person in law.

Finally, to reiterate, there is a growing demand for a tool that could assist procurement, information security and internal developers of AI applications to self-assess a solution and flag if:

— They are performing *low-risk applications* and should go ahead.
— They are performing *medium-risk applications* and should provide more information and implement mitigation strategies.
— They are performing *high-risk applications* and should go through a review process before deploying their solution across business.

We posit that the proposed instrument for this evaluative purpose is algorithm auditing. It is our contention that algorithm auditing, as an integral facet of AI risk management, is poised for exponential growth in the forthcoming decade. This trajectory aligns seamlessly with the increasing emphasis on, and concomitant regulatory and legal imperatives pertaining to, the comprehensive management of risks associated with AI applications.

## Data Availability

This article has no additional data.

## Footnotes

^1^

*L*: loss function; E : expectation operator; *y*: output variable; *x*: input variable; *f*(*x*): algorithm prediction/decision; *p*: sampling distribution of (*x*, *y*); *D^val^*: holdout set of (*x*, *y*); Δ(*x*): set of feasible perturbations (δ) of *x*; *F*: specification mapping *x* and *f*(*x*) in a real number, if *F*(*x*; *f*(*x*)) ≤ 0 then we say it is satisfied; *Sin*(*x^nom^*, δ): the set of all input *x* that are at most 𝛿 distant from *x* ^*nom*^ $\left(Sin\left(x^{nom},\delta \right)=\left\{x:\left\|x-x^{nom}\right\|\infty \leq \delta \right\}\right)$ ; P : probability measure.
